# Microbiological contamination of improved water sources, Mozambique

**DOI:** 10.2471/BLT.22.288646

**Published:** 2022-07-01

**Authors:** Hirotsugu Aiga, Marika Nomura, Mussagy Mahomed, José Paulo M Langa

**Affiliations:** a School of Tropical Medicine and Global Health, Nagasaki University, 1-12-4 Sakamoto, Nagasaki, 852-8523, Japan.; b Japan International Cooperation Agency, Tokyo, Japan.; c Instituto Nacional de Saúde, Ministry of Health, Maputo, Mozambique.

## Abstract

**Objective:**

To assess if water from improved sources are microbiologically safe in Niassa province, Mozambique, by examining the presence of total coliforms in different types of water sources.

**Methods:**

We conducted a cross-sectional household survey in two rural districts of Niassa province during the dry season, from 21 August to 4 October 2019. We observed water sources and conducted microbiological water quality tests and structured household interviews.

**Findings:**

We included 1313 households, of which 812 (61.8%) used water from an improved source. There was no significant difference in presence of total coliforms between water sampled at improved and unimproved water sources, 62.7% (509 samples) and 65.7% (329 samples), respectively (*P*-value = 0.267). Households using improved water sources spent significantly longer time collecting water (59.1 minutes; standard deviation, SD: 55.2) than households using unimproved sources (49.8 minutes; SD: 58.0; *P*-value < 0.001). A smaller proportion of households using improved sources had access to water sources available 24 hours per day than that of households using unimproved sources, 71.7% (582 households) versus 94.2% (472 households; *P*-value < 0.001). Of the 240 households treating water collected from improved sources, 204 (85.4%) had total coliforms in their water, while treated water from 77 of 107 (72.0%) households collecting water from an unimproved source were contaminated.

**Conclusion:**

Current access to an improved water source does not ensure microbiological safety of water and thereby using access as the proxy indicator for safe drinking and cooking water is questionable. Poor quality of water calls for the need for integration of water quality assessment into regular monitoring programmes.

## Résumé

**Objectif:**

Déterminer si les sources d'eau améliorées comportent un risque microbiologique dans la province de Niassa, au Mozambique, en examinant la présence de coliformes totaux dans différents types de sources d'eau.

**Méthodes:**

Nous avons effectué une enquête transversale auprès des ménages dans les districts ruraux de la province de Niassa durant la saison sèche, du 21 août au 4 octobre 2019. Nous avons analysé les sources d'eau, réalisé des tests de qualité microbiologique et mené des entretiens structurés dans les foyers.

**Résultats:**

Nous avons étudié 1313 ménages, dont 812 (61,8%) consommant de l'eau issue d'une source améliorée. En ce qui concerne la présence de coliformes totaux, nous n'avons constaté aucune différence notable entre les sources d'eau améliorées et non améliorées: 62,7% (509 échantillons) pour l'une et 65,7% (329 échantillons) pour l'autre (valeur-p = 0,267). Les ménages utilisant des sources améliorées passaient nettement plus de temps à puiser de l'eau (59,1 minutes; écart type: 55,2) que ceux utilisant des sources non améliorées (49,8 minutes; écart type: 58,0; valeur-p < 0,001). Le pourcentage de ménages exploitant des sources améliorées et ayant accès à ces sources 24 heures sur 24 était plus faible que celui des ménages exploitant des sources non améliorées: 71,7% (582 ménages) contre 94,2% (472 ménages; valeur-p < 0,001). Sur les 240 ménages traitant l'eau prélevée dans des sources améliorées, 204 (85,4%) avaient des coliformes totaux dans leur eau, tandis que l'eau traitée de 77 ménages sur les 107 (72,0%) se procurant de l'eau depuis une source non améliorée était contaminée.

**Conclusion:**

Actuellement, l'accès à une source d'eau améliorée ne permet pas d'écarter tout risque de contamination microbiologique. Par conséquent, l'utiliser comme indicateur indirect pour l'eau potable et l'eau destinée à la préparation des aliments est discutable. La piètre qualité de l'eau met en lumière la nécessité d'intégrer une évaluation de la composition de l'eau dans les programmes de contrôle réguliers.

## Resumen

**Objetivo:**

Determinar si las fuentes de agua mejoradas son seguras desde el punto de vista microbiológico en la provincia de Niassa (Mozambique), mediante el análisis de la presencia de coliformes totales en diferentes tipos de fuentes de agua.

**Métodos:**

Se realizó una encuesta transversal de hogares en dos distritos rurales de la provincia de Niassa durante la estación seca, del 21 de agosto al 4 de octubre de 2019. Se observaron las fuentes de agua y se realizaron pruebas microbiológicas de la calidad del agua y entrevistas estructuradas en los hogares.

**Resultados:**

Se incluyeron 1313 hogares, de los que 812 (61,8 %) utilizaban agua de una fuente mejorada. No hubo diferencias significativas en la presencia de coliformes totales entre el agua obtenida de fuentes de agua mejoradas y no mejoradas, 62,7 % (509 muestras) y 65,7 % (329 muestras), respectivamente (valor de *p* = 0,267). Los hogares que utilizaron fuentes de agua mejoradas emplearon un tiempo significativamente mayor en la recogida de agua (59,1 minutos; desviación estándar, DE: 55,2) que los hogares que utilizaron fuentes no mejoradas (49,8 minutos; DE: 58,0; *p* < 0,001). Un porcentaje menor de hogares que utilizaban fuentes mejoradas tenía acceso a fuentes de agua disponibles las 24 horas del día que el de los hogares que utilizaban fuentes no mejoradas, 71,7 % (582 hogares) frente a 94,2 % (472 hogares; *p* < 0,001). De los 240 hogares que trataban el agua recogida de fuentes mejoradas, 204 (85,4 %) tenían coliformes totales en el agua, mientras que el agua tratada de 77 de 107 (72,0 %) hogares que recogían agua de una fuente no mejorada estaba contaminada.

**Conclusión:**

El acceso actual a una fuente de agua mejorada no garantiza la seguridad microbiológica del agua y, por lo tanto, utilizar el acceso como indicador indirecto de agua potable y para cocinar es cuestionable. La mala calidad del agua exige que se integre la valoración de la calidad del agua en los programas regulares de control.

## ملخص

**الغرض:**

تقييم ما إذا كانت مصادر المياه المحسّنة آمنة من الناحية الميكروبيولوجية في مقاطعة نياسا بموزامبيق، وذلك من خلال فحص وجود بكتيريا القولونية الكلية في أنواع مختلفة من مصادر المياه.

**الطريقة:**

أجرينا مسحًا مقطعيًا أُسريًا في منطقتين ريفيتين في مقاطعة نياسا خلال موسم الجفاف، من 21 أغسطس/آب إلى 4 أكتوبر/تشرين أول 2019. قمنا بملاحظة مصادر المياه وإجراء اختبارات لنوعية المياه الميكروبيولوجية، وإجراء مقابلات أسرية منظمة.

**النتائج:**

قمنا بتضمين 1313 أسرة، منها 812 (61.8%) تستخدم المياه من مصدر محسّن. لم يكن هناك فارق ملموس، في وجود القولونيات الكلية، بين عينات المياه المأخوذة من مصادر المياه المحسّنة وغير المحسّنة، 62.7% (509 عينة)، و65.7% (329 عينة) على التوالي (القيمة *P * ‏= 0.267). ‏قضت الأسر التي تستخدم مصادر مياه محسّنة وقتًا أطول بشكل ملموس في جمع المياه (59.1 دقيقة؛ الانحراف المعياري: 55.2)، مقارنة بالأسر التي تستخدم مصادر غير محسّنة (49.8 دقيقة؛ الانحراف المعياري: 58.0؛ القيمة *P * أقل من 0.001). نسبة الأسر التي تستخدم مصادر محسّنة ولديها إمكانية الوصول إلى مصادر المياه المتاحة طوال 24 ساعة في اليوم، كانت أقل من تلك الأسر التي تستخدم مصادر غير محسّنة، 71.7% (582 أسرة) مقابل 94.2% (472 أسرة؛ القيمة *P * أقل من 0.001). من بين 240 أسرة تعالج المياه التي تم جمعها من مصادر محسّنة، كان لدى 204 أسرة (85.4%) بكتيريا القولونية الكلية في مياههم، بينما المياه المعالجة من 77 من أصل 107 (72.0%) من الأسر التي تجمع المياه من مصدر غير محسّن، كانت ملوثة.

**الاستنتاج:**

إن الوصول الحالي إلى مصدر مياه محسّنة لا يضمن السلامة الميكروبيولوجية للمياه، وبالتالي فإن استخدام الوصول كمؤشر بديل لمياه الشرب والطهي الآمنة، هو أمر مشكوك فيه. تستدعي جودة المياه الرديئة الحاجة إلى دمج تقييم جودة المياه في برامج المراقبة المنتظمة.

## 摘要

**目的:**

旨在通过检测不同水源类型中总大肠杆菌群的含量评估莫桑比克尼亚萨省改善水源的微生物安全性。

**方法:**

我们于旱季期间（2019 年 8 月 21 日至 10 月 4 日）在尼亚萨省两个乡村地区开展了一项横向家庭调查。我们检测了水源并开展了微生物水质检测和结构化家庭访问。

**结果:**

我们纳入 1313 户家庭，其中 812 (61.8%) 户使用改善水源。改善水源和未改善水源水样中的总大肠杆菌群含量无显著差异，分别为 62.7%（509 份样本）和 65.7%（329 份样本）(*P*-value = 0.267)。与使用未改善水源的家庭相比（49.8 分钟；SD：58.0），使用改善水源的家庭在取水方面明显花费更多时间（59.1 分钟；标准偏差，SD：55.2；*P*-value < 0.001）。与使用未改善水源的家庭相比，小部分使用改善水源的家庭可全天 24 小时获取水源，71.7%（582 户家庭）对比 94.2%（472 户家庭；*P*-value < 0.001）。在 240 户采用处理中改善水源的家庭中，204 (85.4%) 户的水源中存在总大肠杆菌群，而在 107 户采用已处理的未改善水源的家庭中，77 72.0%) 户家庭的用水受到了污染。

**结论:**

目前，使用改善水源无法确保水源的生物安全性，因此将使用改善水源作为饮用水和煮饭用水安全的代表性指标是值得怀疑的。由于水质较差，需要将水质量评估整合到常规监测项目中。

## Резюме

**Цель:**

Оценить, являются ли улучшенные источники воды в провинции Ньяса в Мозамбике микробиологически безопасными, путем изучения наличия общих бактерий группы кишечной палочки в различных типах источников воды.

**Методы:**

С 21 августа по 4 октября 2019 года авторы провели поперечно-секционное исследование домохозяйств в двух сельских округах провинции Ньяса в сухой сезон. Авторы наблюдали за источниками воды и проводили микробиологические тесты качества воды, а также систематизированные опросы домохозяйств.

**Результаты:**

Авторы включили в обзор 1313 домохозяйств, из которых 812 (61,8%) использовали воду из улучшенного источника. Не было отмечено существенной разницы в наличии бактерий группы кишечной палочки в целом между пробами воды, отобранной из улучшенных и неулучшенных источников: 62,7% (509 проб) и 65,7% (329 проб) соответственно (P-значение = 0,267). Домохозяйства, использующие улучшенные источники воды, тратили значительно больше времени на сбор воды (59,1 минуты; стандартное отклонение, СО: 55,2), чем домохозяйства, использующие неулучшенные источники (49,8 минут; стандартное отклонение, СО: 58,0; P-значение < 0,001). Среди домохозяйств, использующих улучшенные источники, круглосуточный доступ к источникам воды был возможен для меньшего количества семей, чем среди домохозяйств, использующих неулучшенные источники: 71,7% (582 домохозяйства) по сравнению с 94,2% (472 домохозяйства; P-значение < 0,001). Из 240 домохозяйств, обрабатывающих воду, собранную из улучшенных источников, наличие бактерий группы кишечной палочки (в целом) было выявлено в 204 случаях (85,4%), в то же время среди домохозяйств, получающих воду из неулучшенного источника, обработанная вода оказалась загрязненной в 77 случаях из 107 (72,0%).

**Вывод:**

Существующий доступ к улучшенному источнику воды не гарантирует микробиологическую безопасность воды, следовательно использование такого доступа в качестве косвенного показателя безопасности питьевой воды и воды для приготовления пищи сомнительно. Низкое качество воды свидетельствует о необходимости включения оценки качества воды в программы регулярного мониторинга.

## Introduction

For more than 40 years, ensuring populations’ access to safe drinking water has been on the global agenda. Access to safe drinking water was recognized as a key public health intervention in the Declaration of Alma-Ata in 1978^1^ and the United Nations (UN) International Drinking Water Supply and Sanitation Decade 1981–1990.^2^^,^^3^ More recently the millennium development goal 7 covered access to safe water,^4^ and the sustainable development goal 6 (SDG 6)^5^ – ensure availability and sustainable management of water and sanitation for all – has attracted attention from both governmental and private sectors. 

Since 1990 access to safe drinking water has been monitored through the World Health Organization and United Nations Children’s Fund Joint Monitoring Programme for Water Supply and Sanitation. The definition used for measuring the proportion of the population using safely managed drinking water services is “the proportion of the population using improved drinking water facilities that accesses those facilities with a collection time of 30 minutes or less*”*.^6^ The programme lists piped water, boreholes or tube wells, protected dug wells, protected springs, rainwater and packaged or delivered water as improved water sources.^6^ According to the definition, water from improved sources needs to be free from faecal (total coliforms including *Escherichia coli*) and priority chemical contamination (arsenic and fluoride).^6^ However, the question arises whether water from improved water sources is free from total coliforms. In 2012, the programme proposed integration of water quality testing into nationally representative household surveys. Yet, by 2019, only 29 countries had conducted such surveys.^7^

For example, water quality testing has not been integrated into national household surveys in Mozambique, a country with an estimated 56% of the population with access to improved water sources in 2017.^8^ In rural areas, the coverage is estimated to be only 40%.^9^ Thus, increasing access to improved water sources in rural areas is key to achieving SDG 6 in Mozambique. However, without examining water quality of improved water sources, the efforts of increasing access might be useless. This study aims to assess if water from improved sources is microbiologically safe in two rural districts of Niassa province, Mozambique.

## Methods

To examine the relationship between water source types and water quality, we conducted a cross-sectional household survey in two rural districts of Niassa province, Mozambique, during the dry season, from 21 August to 4 October 2019.

### Study areas

**Fig. 1:**
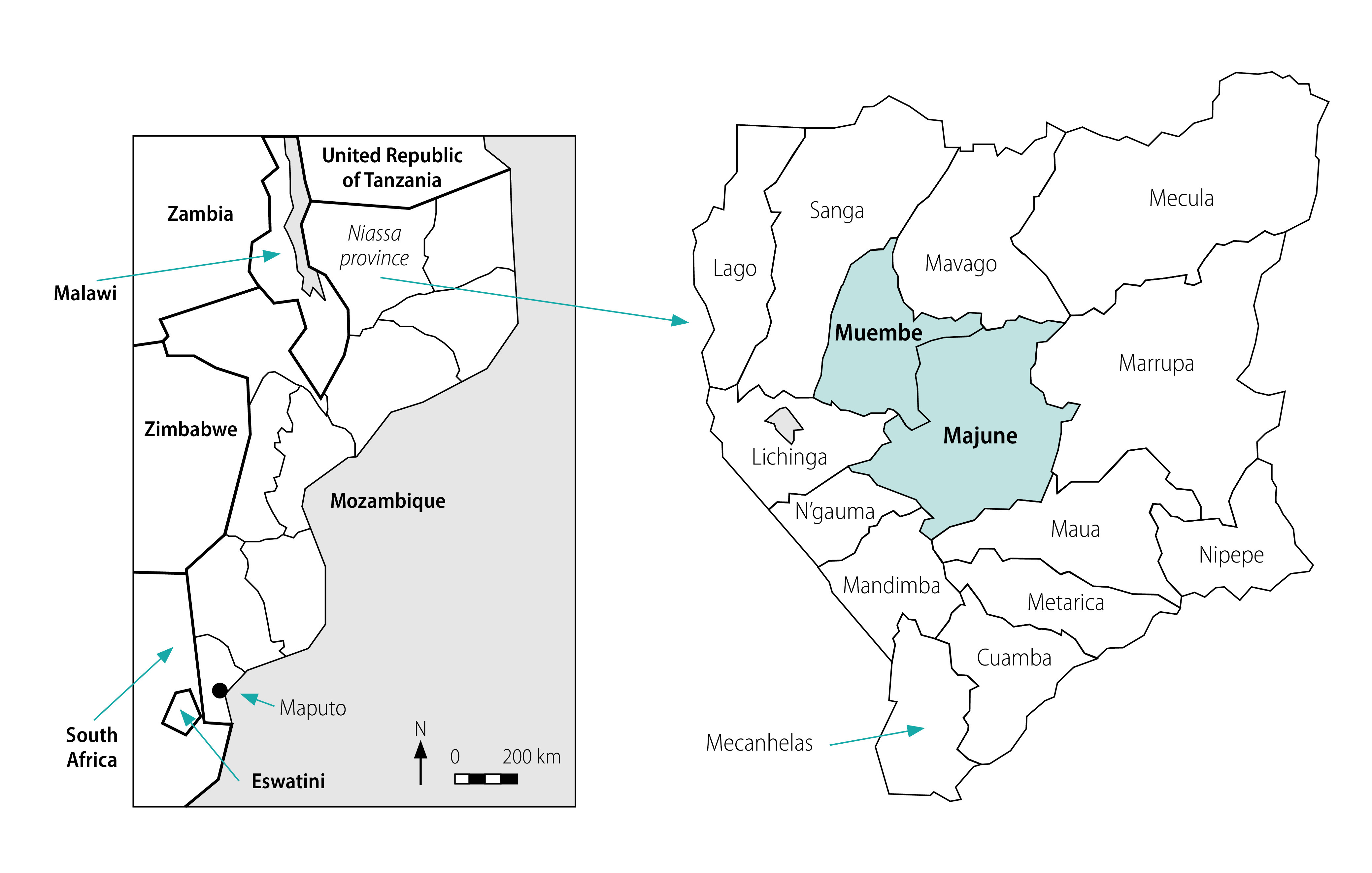
Study area for assessing improved water sources, Niassa province, Mozambique, 2019

Note: The map of Niassa province is adapted from a World Bank map.^10^

Niassa province is located in north-west Mozambique. We focused on two typical rural districts, Majune and Muembe, in Niassa province (Fig. 1).^10^ Majune district is located in the geographical centre of Niassa province, 115 km away from Lichinga, the capital of the province. Muembe district borders with Majune district in the south-east, 84 km away from Lichinga. The two districts are located in the rainy highland (annual precipitation: 1171 mm; altitude: 1500–1600 m).

### Sample size and sampling

In Niassa province, 43.5% of households had access to improved water sources in 2011.^11^ By applying the proportion, the sample size was calculated with an error of 0.05, a power of 0.80 and a precision of 0.05. As the results, we calculated that 776 households was the required sample size. Applying a design effect of 1.8 for two-stage sampling and a non-response rate of 7.5%,^12^ we determined that 1342 households should be the final sample size. Of all 224 enumeration areas in the two districts, we randomly selected 94 by employing systematic random sampling and ensuring probability proportional to size. Then, 1342 households were further randomly selected from the 94 enumeration areas. We visited a household up to three times before we deemed the household unreachable. 

### Water source observation

Household members responsible for water collection and storage guided enumerators to water sources at the typical water collection time of the day. The enumerators confirmed water source type by both directly observing them and cross-checking them with the list of existing water sources (Japan International Cooperation Agency, unpublished data, 2019). We measured time spent collecting water, including: (i) making a round trip to the water source; (ii) waiting in a queue at water sources; and (iii) filling water in containers or buckets. In this study, we defined water collection time as the total number of minutes spent on these three elements of water collection activities. By using the Joint Monitoring Programme’s definitions, we categorized the type of water sources into either improved or unimproved water sources.^6^^,^^9^

### Water sampling

For each household, we collected water samples for microbiological testing at two points: (i) primary source of water for drinking and cooking; and (ii) water vessels, drinking flasks or water dispensers at households. For the households using vendor-provided water, we sampled source water from cart or truck. For those using piped private household connections, we sampled water only from water faucets since we considered the faucets as both a source and household drinking and cooking water.

### Microbiological water quality test

Mozambican national guidelines for drinking water quality require drinking water to be free from total coliforms.^13^ Therefore, we examined presence of total coliforms in the sampled water without quantifying the level of their concentration.^14^ We used SUNCOLI X-type test paper (Kyoritsu Chemical Check Laboratory Co., Tokyo, Japan), which has a sensitivity of 90% and specificity of 80%. After soaking two sheets of the paper in sampled water for three seconds, we kept the soaked papers at 36–37 °C in a non-electric incubator HU-BOX-19–36 (Sanplatec Co., Tokyo, Japan) for 24 hours. After the incubation we examined the colour of the papers and a colour change indicated the presence of total coliforms.

### Household interviews

By using a structured questionnaire, we interviewed household members responsible for water collection and storage about water treatment practices, and household heads about socioeconomic characteristics. We used the Joint Monitoring Programme definition of appropriate water treatments for drinking and cooking.^15^ The structured questionnaire was developed in three locally spoken languages (Ajawa, Macua and Portuguese).

### Data analysis

We entered the data from water source observations, microbiological water quality tests and household interviews into a personal computer. By applying the data on housing materials and ownership of key properties to principal component analysis, we calculated the wealth index for each household. We used the wealth index to categorize all households into wealth quintiles.^16^ We used SPSS for Windows, version 22 (SPSS Inc., Armonk, United States of America) for statistical analyses.

### Ethical considerations

We obtained ethical approval from the Mozambican National Committee for Bioethics in Health (Ref: 279/CNBS/19). The Mozambican Ministry of Health provided official permission for the research implementation. We obtained written informed consent from household members to participate in structured household interviews, and to accompany the study team to water sources and for publication.

## Results

Of 1342 households sampled, 29 (2.2%) could not be reached despite three visits. No household refused to participate. Therefore, a total of 1313 households participated in the study.

Table 1 shows the types of water sources used for drinking and cooking by households. Of all 1313 households, 812 (61.8%) used improved water sources, while 501 (38.2%) used unimproved water sources. The most used water source (620 households; 47.2%) was protected well or borehole with handpump, one of the improved water sources defined by the Joint Monitoring Programme.^6^^,^^9^

**Table 1:** Types of source of water for drinking and cooking used by households, Niassa province, Mozambique, 2019

Type of water source	No. (%) of households
**Improved water source** ^a^	
Piped private household connection	27 (2.1)
Public standpipe	33 (2.5)
Protected well or borehole with handpump	620 (47.2)
Protected well without handpump	128 (9.7)
Protected spring	2 (0.2)
Vendor-provided water (cart and truck)	2 (0.2)
Subtotal	812 (61.8)
**Unimproved water source** ^a^	
Unprotected well	256 (19.5)
Unprotected spring	193 (14.7)
Surface water (river, lake, pond and reservoir)	52 (4.0)
Subtotal	501 (38.2)
**Total**	**1313 (100.0)**

^a^ Classification of water source type is based on the World Health Organization and United Nations Children’s Fund definition.^6^^,^^9^

**Fig. 2:**
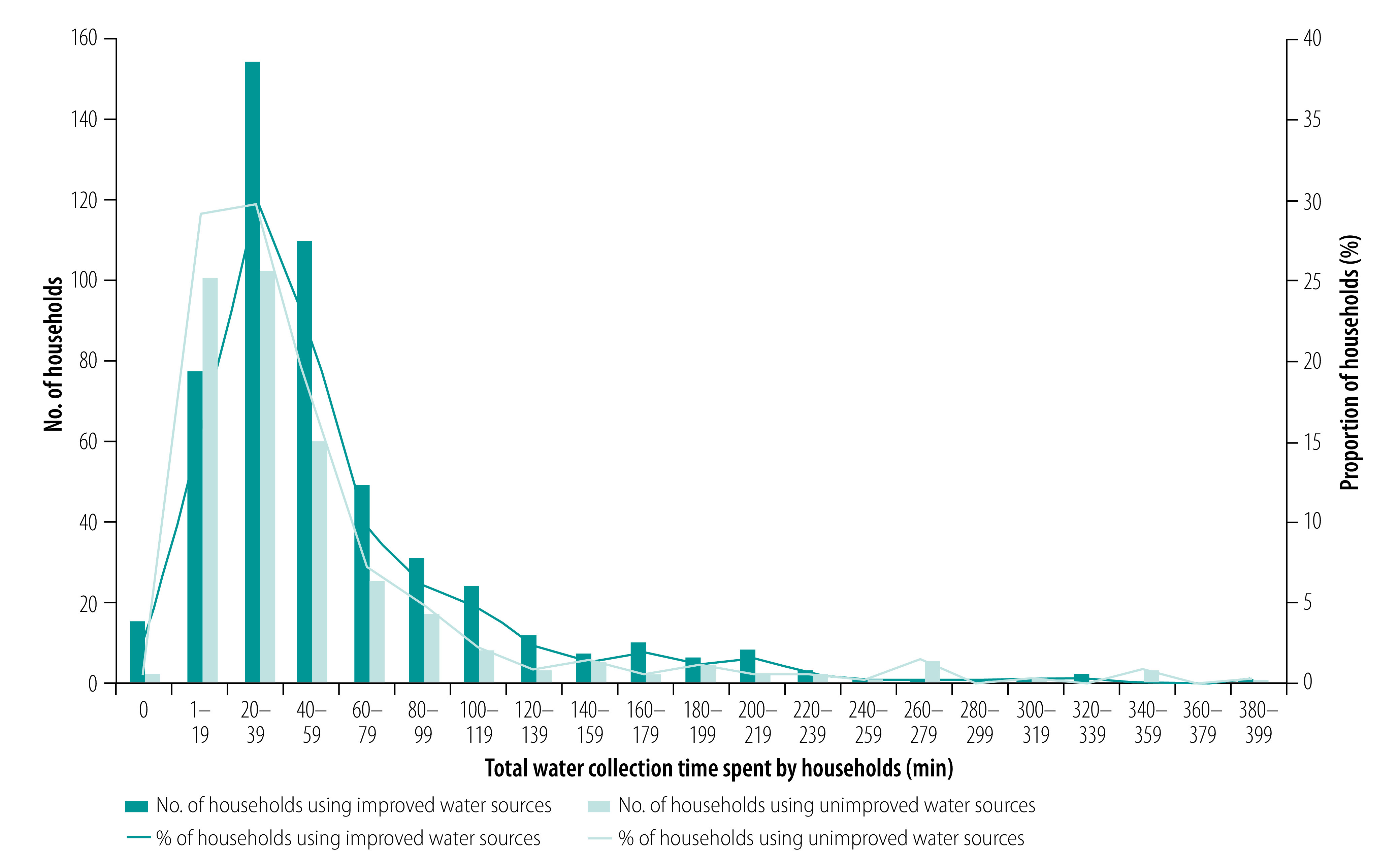
Time spent on water collection by type of water facilities, Niassa province, Mozambique, 2019

Notes: Total water collection time is the sum of time spent: (i) on round trip by routine transport means (on foot or bicycle); (ii) waiting at water sources; and (iii) filling water into containers/buckets. The number of minutes was measured by physically visiting the water source with household members responsible for water collection. We were unable to measure time spent on water collection for 482 of 1278 households (324 of 793 households using improved facilities and 158 of 485 households using unimproved or no facilities), because their accurate routes to water sources were not identified due to absence of household members responsible for water collection when visiting water sources.

Table 2 shows the comparison of access to and quality of water, and households’ socioeconomic characteristics between those using improved water sources and those using unimproved water sources. Total water collection time spent by households using improved water sources (mean: 59.1 minutes; standard deviation, SD: 55.2) was significantly longer than that spent by households using unimproved water sources (mean: 49.8 minutes; SD: 58.0; *P*-value < 0.001; Fig. 2). A smaller proportion of households using improved water sources had access to a water source available 24 hours per day than households using unimproved water sources (71.7%; 582 households versus 94.2%; 472 households; *P*-value < 0.001). Households using improved water sources were more likely to treat their water for drinking and cooking purposes than those using unimproved water sources (30.3%; 246 households versus 21.8%; 109 households; *P*-value = 0.009).

**Table 2:** Access to and quality of water and socioeconomic household characteristics by type of water source, Niassa province, Mozambique, 2019

Characteristic	No. of households (%)^a^		*P*
	Using improved sources of drinking water (*n* = 812)	Using unimproved sources of drinking water (*n* = 501)	
**Socioeconomic**			
No. of household members, mean (SD)	5.59 (2.68)	5.44 (2.20)	0.564^b^
Primary income source			0.204^c^
Agriculture and sales of crops	688 (84.7)	452 (90.2)	
Livestock and sales of animals	2 (0.2)	2 (0.4)	
Fishery	3 (0.4)	1 (0.2)	
Unskilled wage labour	20 (2.5)	8 (1.6)	
Skilled labour	19 (2.3)	4 (0.8)	
Handicrafts and artisanal work	3 (0.4)	2 (0.4)	
Charcoal production	2 (0.2)	2 (0.4)	
Trading and commercial work	31 (3.8)	16 (3.2)	
Salaried worker	40 (4.9)	14 (2.8)	
Government allowance (pension, disability benefit and other social support)	3 (0.4)	0 (0.0)	
Begging and dependent on assistance	1 (0.1)	0 (0.0)	
Wealth quintile^d^			< 0.001^c^
First	158 (19.5)	113 (22.6)	
Second	143 (17.6)	123 (24.6)	
Third	172 (21.2)	96 (19.2)	
Fourth	149 (18.3)	102 (20.4)	
Fifth	190 (23.4)	67 (13.4)	
**Water**			
Total water collection time in min, mean (SD)^e^	59.1 (55.2)	49.8 (58.0)	< 0.001^b^
Water availability at water source			< 0.001^c^
24 hours a day	582 (71.7)	472 (94.2)	
On and off (available only when public water attendant is on duty)	203 (25.0)	25 (5.0)	
Don’t know	27 (3.3)	4 (0.8)	
Water quality at water source^f^			0.267^c^
Presence of total coliforms	509 (62.7)	329 (65.7)	
Absence of total coliforms	284 (35.0)	156 (31.1)	
Not tested due to unavailability of water at source	19 (2.3)	16 (3.2)	
Water treatment for drinking and cooking^g^			0.009^c^
Treatment	246 (30.3)	109 (21.8)	
No treatment	566 (69.7)	392 (78.2)	
Water quality at household^f^			0.153^c^
Presence of total coliforms	611 (77.0)	356 (73.4)	
Absence of total coliforms	160 (20.2)	119 (24.5)	
Not tested due to unavailability of vessels/drinking flasks/water dispensers	22 (2.8)	10 (2.1)	

^a^ If not otherwise stated values are no. (%).

^b^ Mann–Whitney’s U test.

^c^ χ^2^ test or Fisher’s exact test.

^d^ First quintile is the poorest population and the fifth quintile the richest.

^e^ Sum of time spent: (i) on round trip by routine transport means (on foot or bicycle); (ii) waiting at water sources; and (iii) filling water into containers/buckets. The number of minutes was measured by physically visiting water source with household members responsible for water collection.

^f^ For those using piped private household connection, we considered the water quality at source the same as at household.

^g^ Types of water treatment for drinking and cooking include: (i) boiling water; (ii) adding bleach or chlorine; (iii) water filtering; (iv) solar disinfection; and (v) stand and settle for 30 minutes or longer.^15^

We did not detect any significant difference in household size (*P*-value = 0.564) and type of primary income sources (*P*-value = 0.204) between households using improved water sources and those using unimproved water sources. Nevertheless, the proportions of the richest and richer wealth quintiles were greater among households using improved water sources (23.4%; 190 households and 18.3%; 149 households, respectively) than among those using unimproved water sources (13.4%; 67 households and 20.4%; 102 households, respectively; *P*-value < 0.001).

There was no significant difference in the presence of total coliforms in sampled water between improved and unimproved water sources; 62.7% (509 samples) and 65.7% (329 samples), respectively (*P*-value = 0.267). However, the presence of total coliforms in water sampled at households using improved water sources (77.0%; 611 households) was higher, but not significantly, than that of water sampled at households using unimproved water sources (73.4%; 356 households; *P*-value = 0.153). 

**Fig. 3:**
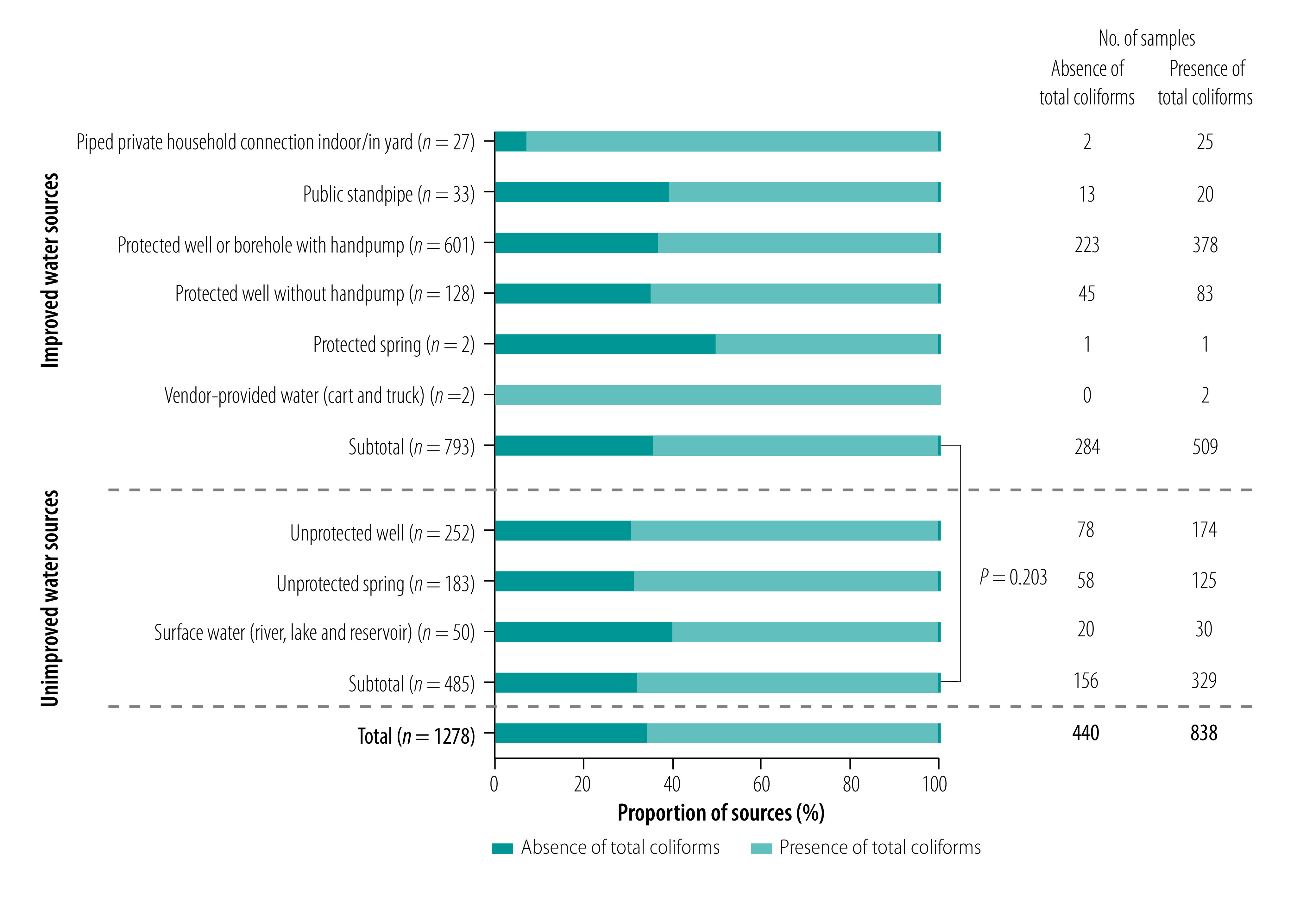
Prevalence of total coliforms in water by type of water source, Niassa province, Mozambique, 2019

Notes: Classification of water source types is based on the updated World Health Organization and United Nations Children’s Fund.^6^^,^^9^ We calculated the *P*-value by using χ^2^ test. We were unable to measure water quality for 35 households, due to unavailability of water upon the visits to water sources. Therefore, the total number of households and *P*-value for *χ*^2^ test are slightly different from those in Table 2.

The proportion of samples containing total coliforms is shown by type of water source in Fig. 3. Overall proportion of samples from water sources containing total coliforms was 65.6% (838/1278). This proportion did not differ between improved and unimproved water sources (*P*-value = 0.203). Surprisingly, we detected total coliforms in water sampled at 25 of 27 (92.6%) piped private household connections. Moreover, in the most common water source used, that is protected wells or boreholes with handpumps, we detected total coliforms in water sampled from 62.9% (378/601) of sources. One of the two protected springs sampled contained total coliforms, and total coliforms were present in both samples from vendor-provided water. For unimproved water sources, the proportion of the presence of total coliforms varied from 60.0% (30/50) in surface water to 69.0% (174/252) in unprotected wells.

**Fig. 4:**
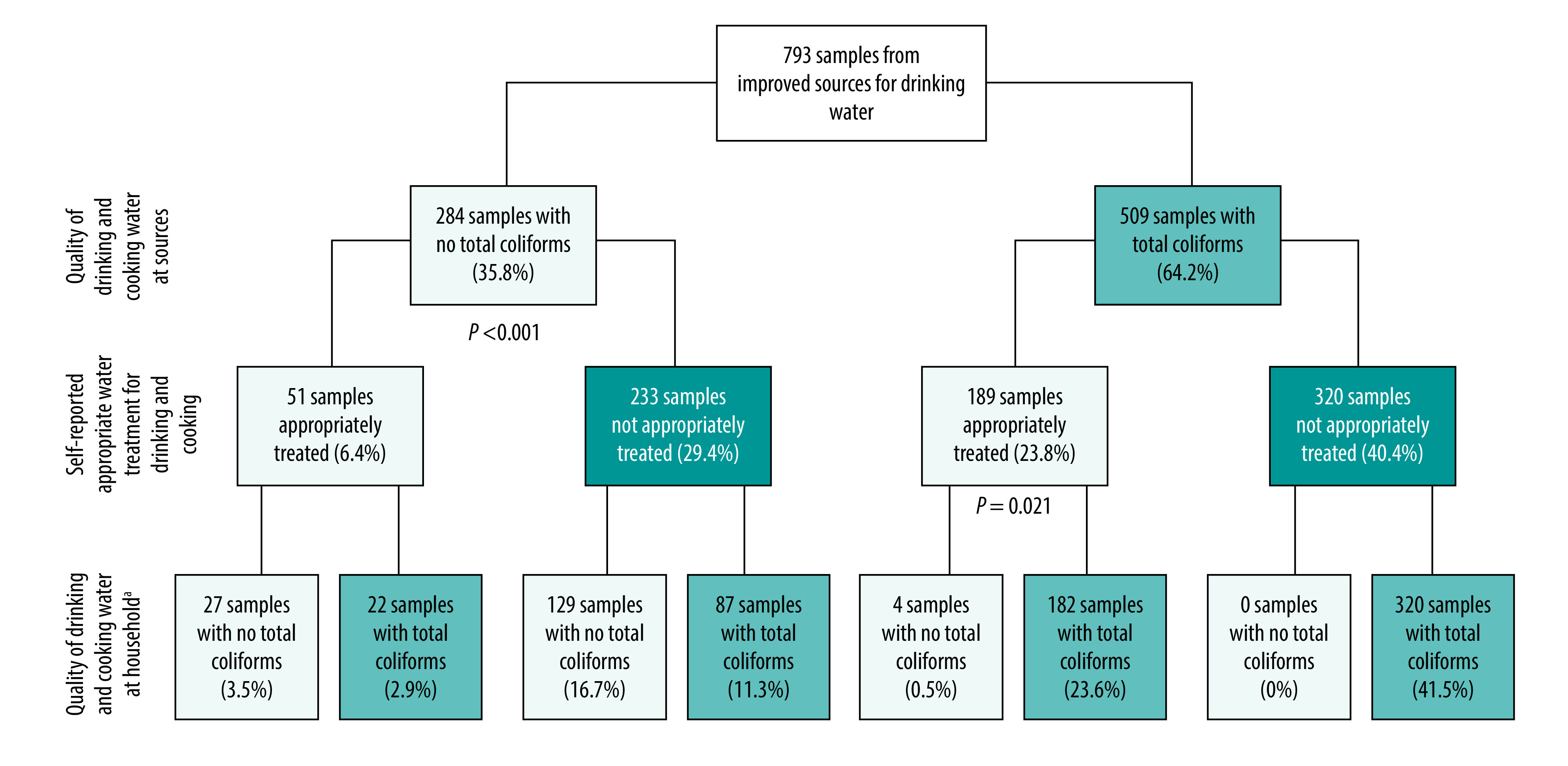
Microbiological water quality and water treatment practices at households using improved water sources, Niassa province, Mozambique, 2019

^a^ Out of the 793 households, we were unable to test the water quality at 22 households.

Notes: Improved facilities for water source include: (i) piped private household connection indoor/in yard; (ii) public standpipe; (iii) protected well/borehole with handpump; (iv) protected well without handpump; (v) protected spring; and (vi) vendor-provided water such as cart and truck.^6^^,^^9^ We were unable to measure water quality for 19 households due to unavailability of water upon the visits to water sources. Types of appropriate water treatment include: (i) boiling water; (ii) adding bleach or chlorine; (iii) water filtering; (iv) solar disinfection; and (v) stand and settle for 30 minutes or longer.^15^ We calculated *P*-values using *χ*^2^ tests.

Of the 240 households appropriately treating their water collected from improved water sources, 204 (85.0%) had total coliforms in their drinking water regardless of the water quality at the source. A significantly higher proportion of households (37.1%; 189/509) with total coliforms in the water source appropriately treated their water than households with no total coliforms in the source (18.0%; 51/284; *P*-value < 0.001; Fig. 4). For the 189 households that treated their water collected from a contaminated source, only four (2.1%) households had no coliforms in their drinking and cooking water (Fig. 4). 

**Fig. 5:**
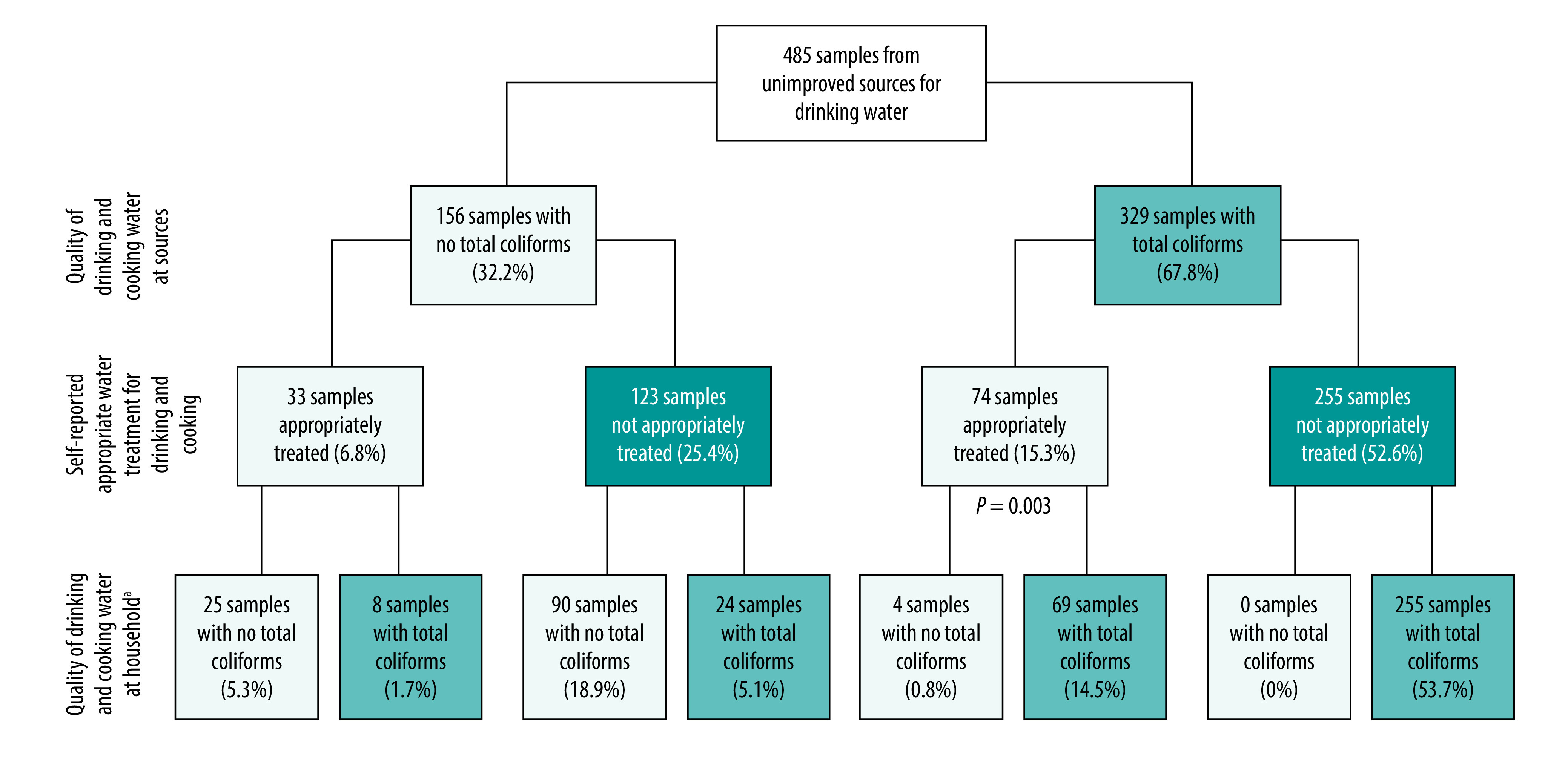
Microbiological water quality and water treatment practices at households using unimproved water sources, Niassa province, Mozambique, 2019

^a^ Out of the 485 households, we were unable to test the water quality at 10 households.

Notes: Unimproved or no facilities for water source include: (i) unprotected well; (ii) unprotected spring; and (iii) surface water (river, lake, reservoir).^6^^,^^9^ We were unable to measure water quality for 16 households due to unavailability of water upon the visits to water sources. Types of appropriate water treatment include: (i) boiling water; (ii) adding bleach or chlorine; (iii) water filtering; (iv) solar disinfection; and (v) stand and settle for 30 minutes or longer.^15^ We calculated *P*-values using *χ*^2^ tests.

Of the 107 households treating their water from an unimproved water source, 72.0% (77 households) had total coliforms in their drinking water (Fig. 5). This proportion was lower than for households not practising water treatment (73.8%; 279/378). However, only four households treating contaminated water from source had drinking and cooking water without total coliforms (Fig. 5). 

## Discussion

Here we show that water from many improved sources in Niassa province, Mozambique are of poor quality. Some explanations for this result could be inadequate water treatment at production stage or lack of cleaning of water tanks located on the roofs of houses, which will affect the quality of piped private water. Similarly, water containers or tanks that water vendors use have probably not been cleaned enough. Poor quality of water from these types of improved water sources was reported by several earlier studies in low- and middle-income countries such as Cambodia,^17^ Dominican Republic^18^ and Ethiopia.^19^^,^^20^ On the other hand, we show that 40% of surface water was not contaminated with total coliforms. The high percentage could be explained by the data collection happening during the dry season, since total coliforms in surface water may be lower in the dry season than in the rainy season.^21^ Hence, a year-round water quality examination needs to be conducted for all types of water sources. In general, the design of protected wells or boreholes with handpumps is better to produce adequate quality and quantity of water all year around, while some unprotected wells or springs could produce poor quality and quantity of water and could even be dried up in the dry season. In addition, households’ water collecting behaviours need to be studied for the entire year, because a certain proportion of households might switch water sources between dry and rainy seasons.

When being constructed, all the improved water sources in Majune and Muembe districts were microbiologically and chemically examined to meet the drinking water quality requirement specified in the Mozambican national guidelines (that is, no total coliforms per 100 mL of water).^13^ Yet, chronic financial and human resource constraints have been reported to prevent provincial and district health directorates from regularly monitoring water quality at all the improved water facilities.^22^ As a result, only improved water sources that serve greater populations in the districts are selectively targeted for regular water quality monitoring.^22^ Thus, quality of water from many improved sources remain neither monitored nor examined. Despite existing policies on regular water quality monitoring and long-term water source maintenance and rehabilitation planning,^23^ their implementation is likely to have been interrupted, irregular and less systematic. Probably, this operational issue has made the microbiological quality of water from improved sources equally as poor as that from unimproved sources.

We found that those using improved water sources were willing to spend significantly longer time accessing and collecting water that is significantly less likely to be available for 24 hours a day. In the districts, a household using improved water sources is charged on average 16.5 Mozambican metical (equivalent to 0.264 United States dollars) per month, regardless of the amount of water they collect and household size.^23^ Those payments are pooled for regular and irregular maintenance of water source facilities. The monthly charge is equivalent to 0.41% of monthly household income,^23^ much lower than the water affordability threshold set by the UN Department of Economic and Social Affairs (3% of household income).^24^ Thus, water from improved sources should be affordable in both Majune and Muembe districts, implying that some residents might have been refraining from using improved water sources not for financial reasons but for other reasons, such as longer water collection time.

In addition, those using improved water sources are not only spending more temporal and financial resources, but also are more frequently practising water treatment than those households using unimproved water sources. It might be assumed that those using water from unimproved sources should be more motivated to treat water, if they are aware that the water they collect could be contaminated. One of the possible reasons for local residents to select improved water sources is the level of their water quality consciousness. The more conscious people are about water for drinking and cooking, the more often they treat water to be stored in vessels, drinking flasks and water dispensers. This finding is in line with an earlier study on household water treatment behaviours in Indonesia.^25^ Only about 3% of those treating contaminated water, regardless of source, were successful in removing total coliforms, probably due to inadequate treatment, such as insufficient boiling time or chlorine concentration. Also, some of those self-reporting practising water treatments might not have practised it in reality. On the other hand, those using water from unimproved sources might even have been unaware that they used contaminated water. This study did not assess whether each household was aware of which category of water source type it was using (that is, improved water source or unimproved water source). Thus, we cannot discuss further households’ water-related behaviours.

This study has several limitations. First, microbiological water quality tests were conducted only for presence or absence of total coliforms. Second, we did not do a chemical or radiological assessment of the water quality. Third, the cross-sectional data between August and October have limited representativeness to the entire year. Fourth, generalizability of the study results is limited, as the findings were based exclusively on the data collected in two districts. 

To identify the relationship more precisely between water source types and water quality, microbiological water quality tests should be conducted not just by examining presence of total coliforms but also by quantifying the levels of contamination (e.g. the most probable number of total coliforms per 100 mL). However, challenges in locally setting up a laboratory-based water quality testing system in rural areas must be recognized and overcome by national and local authorities. Otherwise, integration of water quality testing into research and regular monitoring would not be realized. Moreover, a combination of microbiological and chemical quality tests (e.g. total coliforms and free residual chlorine) should be conducted as the standard package to assess broader aspects of water quality.

As of 2017, a total of 133 protected wells or boreholes with handpumps in Majune and Muembe districts were registered in Niassa Provincial Public Works Department (Japan International Cooperation Agency, unpublished data, 2019). Most of them (104; 78.2%) were constructed through external financial supports (e.g. African Development Bank, Irish Aid, Japan International Cooperation Agency, Swiss Agency for Development Cooperation and nongovernmental organizations), while the rest were constructed by provincial departments of public works. Despite tremendous external support for construction of improved water sources, water quality monitoring has been sporadic, inadequate and irregular due to chronic budgetary constraints at provincial health departments responsible for water quality monitoring. In view of the current challenging circumstances, we recommend that external development agencies provide post-construction supplementary or counterpart funding for water quality monitoring activities. This funding will help enhance and sustain integration of water quality assessment into regular monitoring, through collaboration among all the actors (government, UN, bilateral agencies and nongovernmental organizations). Water quality assessment and nationally representative household surveys^7^ should be part of regular water source monitoring.

Globally, the proportion of those having access to improved sources of water increased from 82% in 2000 to 93% in 2020.^9^ Now that more people have access to improved water sources, greater attention should be paid to the quality of water. Access to improved water source does not ensure microbiological safety of water and thereby using access as the proxy indicator for safe drinking water is questionable. Poor quality of water, regardless of type of water source, calls for integration of water quality assessment into regular monitoring programmes.
